# Deciphering the Genetic Architecture of *Staphylococcus warneri* Prophage vB_G30_01: A Comprehensive Molecular Analysis

**DOI:** 10.3390/v16101631

**Published:** 2024-10-19

**Authors:** Fangxiong Pu, Ning Zhang, Jiahe Pang, Nan Zeng, Faryal Babar Baloch, Zijing Li, Bingxue Li

**Affiliations:** 1 College of Bioscience and Biotechnology, Shenyang Agricultural University, Shenyang 110866, China; pufangxiong@gmail.com (F.P.); pangjiahe2023@163.com (J.P.); 2 College of Land and Environment, Shenyang Agricultural University, Shenyang 110866, China; zengnan1015@163.com (N.Z.); faryalbabarbaloch@gmail.com (F.B.B.); 3 Food Science College, Shenyang Agricultural University, Shenyang 110866, China; 15002492493@163.com

**Keywords:** CNS prophage, *Staphylococcus warneri*, genomic analysis, transcriptional regulator

## Abstract

The current knowledge of *Staphylococcus warneri* phages is limited, with few genomes sequenced and characterized. In this study, a prophage, vB_G30_01, isolated from *Staphylococcus warneri* G30 was characterized and evaluated for its lysogenic host range. The phage was studied using transmission electron microscopy and a host range. The phage genome was sequenced and characterized in depth, including phylogenetic and taxonomic analyses. The linear dsDNA genome of vB_G30_01 contains 67 predicted open reading frames (ORFs), classifying it within Bronfenbrennervirinae. With a total of 10 ORFs involved in DNA replication-related and transcriptional regulator functions, vB_G30_01 may play a role in the genetics and transcription of a host. Additionally, vB_G30_01 possesses a complete set of genes related to host lysogeny and lysis, implying that vB_G30_01 may influence the survival and adaptation of its host. Furthermore, a comparative genomic analysis reveals that vB_G30_01 shares high genomic similarity with other *Staphylococcus* phages and is relatively closely related to those of *Exiguobacterium* and *Bacillus*, which, in combination with the cross-infection assay, suggests possible cross-species infection capabilities. This study enhances the understanding of *Staphylococcus warneri* prophages, providing insights into phage–host interactions and potential horizontal gene transfer.

## 1. Introduction

Viruses that infect bacteria are called phages, which can be classified into virulent phages and temperate phages. Temperate phages, integrated into the bacterial genome or persisting as low-copy-number plasmids, are known as prophages [1]. In addition to protecting host bacteria from invasion by other phages, prophages also act as vehicles for facilitating horizontal gene transfer [2]. Numerous investigations have demonstrated that bacteriophage-encoded antibiotic resistance genes (ARGs) can enhance corresponding resistance in host bacteria, serving as a key driver of antibiotic resistance gene transfer in nature [3]. Additionally, phages may carry potential virulence factors (VFs) [2], promoting bacterial adhesion, colonization, evasion of immune systems, and serum resistance, among other functions [4].

Staphylococci, a type of Gram-positive bacteria commonly found on human and animal skin, in oral cavities, and various other areas [5], is classified based on its ability to coagulate rabbit blood into coagulase-positive and -negative groups, with coagulase considered a major virulence factor [6]. Coagulase-negative staphylococci (CNS), which are typically lacking pathogenicity, are categorized as minor pathogens that are widely distributed in the natural environment, with significant genetic diversity [7]. Due to variations in pathogenicity and clinical significance among different CNS species, they should be considered separately. *Staphylococcus warneri* (*S. warneri*), a common conditional pathogen among CNS in clinical settings [8], has the potential to cause endocarditis [9], sepsis [10], suppurative arthritis [11], spinal osteomyelitis [12], meningitis, and neonatal infections [13,14], particularly in individuals with compromised immunity. Moreover, *S. warneri* might be significantly associated with subclinical mastitis in dairy cows [15,16].

The predilection of *Staphylococcus* to infect humans or animals may be governed by factors encoded in phages integrated into the bacterial genome [17]. Studies have identified the presence of multidrug-resistant strains of *Staphylococcus warneri*, including resistance to antibiotics such as vancomycin, teicoplanin, cefoxitin, and tetracycline [18,19,20]. The use of specialized and personalized phage mixtures has been proven to be a viable alternative for combating multidrug-resistant bacteria. Furthermore, phage therapy for coagulase-negative staphylococci infections is already under-researched [21]. It is worth mentioning that in addition to virulent phages, temperate phages might serve as substitutes for antibiotics [22], with successful instances of eradicating lysogenic bacteria through their combined use with antibiotics [23]. In the NCBI database, only a few genomes of CNS phages have been sequenced and characterized compared to those of *Staphylococcus aureus* [24,25,26,27,28,29]. Comprehensive genomic analyses of *Staphylococcus warneri* phages are virtually unreported.

In this investigation, we reported an inducible prophage vB_G30_01 from *Staphylococcus warneri* strain G30 that was isolated from cherry rhizosphere soil. Subsequently, the phage underwent a morphological identification and comprehensive genomic analysis. This study marks the inaugural report in public databases concerning the genome and comparative genomics of a *Staphylococcus warneri* phage [30,31]. The findings not only enhance our comprehension of temperate phages in *Staphylococcus warneri* but also contribute to elucidating the evolution of the *Staphylococcus* phage family. Moreover, these results establish a theoretical foundation for understanding the phage-mediated transfer of resistance and virulence genes, the modification of host bacterial functions, and the potential application of phage therapy.

## 2. Materials and Methods

### 2.1. Host Strain and Culture Conditions

The host strain, *Staphylococcus warneri* G30, was isolated from cherry rhizosphere soil. Soil suspension was spread on solid plates containing 2% agar beef extract medium, and single colonies were selected and purified through repeated streak plate methods. The purified strain was then inoculated in beef extract liquid medium and incubated at 37 °C with 180 rpm shaking for 12 h. Subsequently, it was transferred to tubes, with a final glycerol concentration of 25% for storage at −80 °C.

### 2.2. Isolation of Prophage

A new prophage, vB_G30_01, was induced from *Staphylococcus warneri* G30 using mitomycin C. The experimental procedure is outlined as follows: *Staphylococcus warneri* G30, cultured to the logarithmic growth phase, underwent treatment with 1 μg.μL^−1^ of mitomycin C and was then incubated at 37 °C for 12 h. Following incubation, the culture was centrifuged at 12,000 rpm for 20 min, and the supernatant was sequentially filtered through 0.45 μm and 0.22 μm micropore filters. A preliminary concentration with polyethylene glycol 8000 was applied to obtain a crude phage particle extract. Subsequently, a CsCl gradient solution was prepared, added to centrifuge tubes from high to low density, topped with the phage crude extract, and centrifuged at 100,000× *g* for 3 h at 4 °C in an ultracentrifuge. The faint blue band of phage liquid was gradually drawn out with a syringe, and the obtained phage particle concentrate was further purified from CsCl using a 100 kDa ultrafiltration tube and then stored at 4 °C for subsequent experiments.

### 2.3. Morphological Observation

The phage particle morphology was examined using a transmission electron microscope. Purified phage samples were deposited on copper grids, negatively stained with phosphotungstic acid, and observed under a transmission electron microscope at 100 kV to determine the phage size.

### 2.4. Host Range

Cross-infection experiments were performed with various strains of *Staphylococcus*, *Burkholderia*, and *Bacillus* that were available in the laboratory. A spot assay was employed to assess the ability of phage vB_G30_01 to infect other bacteria, thus establishing its host range. Following the gradient dilution of the phage particle stock, the spot inoculation method was applied to double-layer agar plates containing different host bacteria. The base layer, consisting of 1.5% agar, supported the top layer of 0.5% semi-solid agar medium. Then, 2.5 μL of phage particle stock was inoculated, and phenotypes were observed at 24 and 48 h to determine phage infection based on the lysis of bacterial lawns on the plates.

In cases in which no phage plaques were observed on the plates, additional investigations were carried out to assess the lysogenic potential of vB_G30_01 in the test strains, as described by Zheng et al. [32], with modifications. Briefly, each host bacterium was cultured to the logarithmic growth phase in beef extract liquid medium and incubated at 37 °C with 180 rpm shaking. *Staphylococcus warneri* G30 was used as the positive control group. The treatment group received concentrated vB_G30_01, while the control group received a blank beef extract liquid medium. The phage was incubated with the host bacteria at the optimal temperature for 30 min to facilitate infection. After centrifugation at 5000 rpm for 10 min, the supernatant was removed, and the pellet was washed with fresh culture medium. Centrifugation at 5000 rpm for another 5 min was performed after each wash and repeated three times. Each sample was then inoculated into a fresh liquid culture medium for shaker incubation, and cell samples were collected at 0, 3, 6, 9, and 12 h. The bacterial pellet was rapidly frozen with liquid nitrogen and stored at −80 °C. The entry and replication of vB_G30_01 within bacterial cells were assessed by detecting the presence and copy variation of the major capsid protein gene (*mcp*) of vB_G30_01. Primers for the detection of *mcp* were designed using Primer 5. PCR-F (5′-ATGGCTACATTAGACGAACAAGCAA-3′) and PCR-R (5′-TTATGCAGTTGGCTCTGAACCTGT-3′) were designed for presence detection. The DNA of the sample was extracted using a Rapid Bacterial Genomic DNA Isolation Kit (Sangon Biotech, Shanghai, China). The reaction system and amplification conditions are shown in Table S1. While qPCR-F (5′-ATTTTAGCGGCTTACATGCGTTTCG-3′) and qPCR-R (5′-GCTCTGAACCTGTGCCTAAATCTCC-3′) were designed for the copy quantification of *mcp* using an SYBR green-based quantitative real-time PCR. Sample RNA was extracted using a Spin Column Bacteria Total RNA Purification Kit (Sangon Biotech, Shanghai, China) and reversed to cDNA for qPCR. The reaction system and amplification conditions are shown in Table S2. Temperate phages that adsorbed and injected DNA into the cell may exist at low copy numbers or integrate onto the host genome to co-replicate where the mcp expression also rises. Therefore, the *mcp* copy number was quantified to determine whether vB_G3_01 adsorbed into or replicated within the cell.

### 2.5. Extraction and Identification of Phage DNA

Phage DNA was extracted using the phenol–chloroform method, dissolved in sterile water, and stored at −20 °C. Subsequently, the phage DNA underwent gel electrophoresis using low-concentration agarose (Lonza, Basel, Sweden) in a horizontal electrophoresis apparatus. Phage DNA samples mixed with 6× loading buffer in a ratio of 1:5 were electrophoresed on a 0.3% agarose gel at 3 V·cm^−1^ for three hours, followed by analysis using a gel imager.

### 2.6. Phage Genome Sequencing and Analysis

The genome sequencing process was composed of quality control, library construction, and sequencing stages. Initially, the total DNA quantity was assessed using the Quant-iT PicoGreen dsDNA Assay Kit fluorescent dye. Subsequently, a phage library was prepared for Illumina sequencing with the TruSeqTM DNA Sample Prep Kit. Sequencing was conducted on the Illumina NovaSeq platform, employing a whole-genome shotgun strategy for paired-end sequencing of the library. AdapterRemoval (v2.2.2) [33] was utilized to eliminate excess adapters from the 3’ ends, while SOAPec (v2.03) [34] was applied for the quality correction of all reads based on Kmer frequency. A5-MiSeq (v20160825) [35] and SPAdes (v3.12.0) [36] were then employed for de novo assembly of the curated sequencing data to generate contigs. Sequences were extracted based on the sequencing depth of the assembled sequences, and those with high sequencing depths were aligned against the NT database on NCBI using blastn [37] to select viral genome sequences from the assembly results. Finally, results from different software were collated for a collinearity analysis, using MUMmer (v3.1) [38] to determine the positional relationship between contigs and fill gaps between them. Pilon (v1.18) [39] was applied to correct the results.

### 2.7. Data Analysis

The nucleotide similarity of the phage was assessed using blastn in the NCBI database (https://blast.ncbi.nlm.nih.gov/Blast.cgi) accessed on 1 December 2022. PHASTER (https://phaster.ca/) accessed on 1 December 2022 [40] was employed to predict phage regions in bacterial genomes, while GeneMarkS (v4.32) [41] was utilized for open reading frame (ORF) predictions. Diamond (v0.8.36), which is based on the NR database [42], facilitated the alignment of protein-coding gene sequences. Furthermore, HHpred (https://toolkit.tuebingen.mpg.de/tools/hhpred) accessed on 2 December 2022, employing hidden Markov models to scan extensive protein databases, was used to predict protein functions more accurately [43]. For the prediction of potential antibiotic resistance genes, the comprehensive antibiotic research database (CARD) (https://card.mcmaster.ca/) accessed on 14 December 2022 [44] was consulted. Virulence genes were predicted using the Virulence Factors Database (VFDB) (http://www.mgc.ac.cn/VFs/) accessed on 14 December 2022 [45]. Phage similarity was calculated through VIRIDIC (v1.1) (http://rhea.icbm.uni-oldenburg.de/VIRIDIC/) accessed on 15 December 2022 [46]. Easyfig 2.2.3 [47] was employed to conduct a comparative genomic analysis of phages. Sequences were aligned using mafft (https://mafft.cbrc.jp) accessed on 20 December 2022 [48]. Neighbor-joining methods were applied to construct major capsid protein and terminase large subunit phylogenetic trees with regular bootstrap in 1000 replicates using IQ-TREE2 (v2.2.0) [49]. The best match model is LG + F + G4 and VT + F + I + G4.

## 3. Results

### 3.1. Isolation and Biological Characteristics of Prophage vB_G30_01

**Figure 1:**
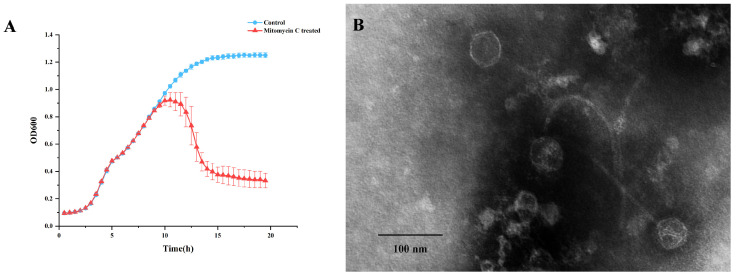
(**A**) Growth curve of *Staphylococcus warneri* G30. The blue curve represents the control group, while the red curve indicates the group treated with 1 µg·ml^−1^ mitomycin C after 6 h of cultivation. (**B**) Transmission electron microscopy image of *Staphylococcus warneri* phage vB_G30_01 magnified at 40,000×, with a scale bar of 100 nm.

The *Staphylococcus warneri* strain G30 was isolated from cherry rhizosphere soil. Upon induction with 1 µg.ml^−1^ mitomycin C, a significant decline in bacterial growth was observed in the culture of this strain (Figure 1A). The phage was subsequently extracted from the supernatant and designated as vB_G30_01. The host range determination of vB_G30_01 was performed on 10 bacteria strains, with no clear plaques observed (Table S3). Transmission electron microscopy revealed that phage vB_G30_01 possesses a virion head approximately 49 nm in diameter and a long, noncontractile, flexible tail measuring about 220 nm in length (Figure 1B).

Following phage adsorption, positive results were observed in gel electrophoresis for the PCR amplification product of the capsid protein nucleotide sequence of phage vB_G30_01 in *Staphylococcus warneri* G30, *Bacillus velezensis* D103, and *Staphylococcus epidermidis* G28, with distinct bands appearing near 1000 bp (refer to Figure S1). *S. warneri* G30, serving as the positive control, showed the brightest concentration band. Conversely, the PCR amplification products for the control groups of these other two bacterial strains yielded negative results in gel electrophoresis, with no visible bands (Figure S2).

**Figure 2:**
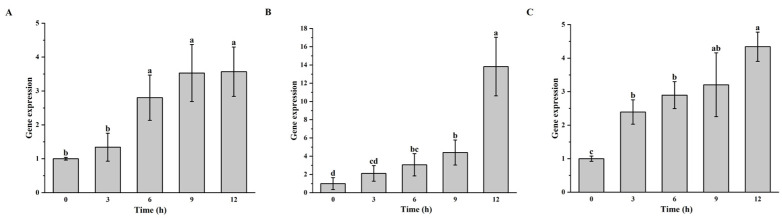
Gene expression level of *mcp* in host bacteria after phage vB_G30_01 infestation at 0, 3, 6, 9, and 12 h. (**A**) *S. warneri* G30 served as the positive control group; (**B**) *S. epidermidis* G28; (**C**) *B. velezensis* D103. Different letters (a–d) indicate the degree of significant difference between treatments. Values with the same letter above the bar graph indicate no significant difference according to the Tukey test.

Real-time fluorescence quantitative PCR was conducted on the culture products of *S. warneri* G30, *B. velezensis* D103, and *S. epidermidis* G28 at 0, 3, 6, 9, and 12 h post-infection with vB_G30_01. The results indicated in Figure 2A show that the expression of *mcp* in the control *S. warneri* G30 showed an increase at 0, 3, 6, and 9 h, with a significant increase at 6 h that leveled off by 9 h. Figure 2B shows that *S. epidermidis* G28 showed an increase in *mcp* gene expression in samples at all time periods after phage treatment. It was significantly increased from 6 h compared to 0 h, while it was also significantly increased at 12 h compared to 9 h. Figure 2C shows that a significant increase in *mcp* gene expression was observed in samples of *B. velezensis* D103 after phage treatment at 3 h, followed by a sustained increase at 6, 9, and 12 h.

### 3.2. Analysis of the Genome of vB_G30_01

The genome of the phage vB_G30_01 is a linear DNA strand comprising 41,707 base pairs, with a GC content of 33.54%. The bases of all mapped reads are about 2.6 G, resulting in a sequencing depth of 64,000×. It contains 67 predicted open reading frames (ORFs) (Table S4), totaling 38,208 base pairs, which account for 91.61% of the entire genome. The average length of an ORF is 570.27 base pairs, resulting in a gene density of 1.61 genes per kilobase and an average GC content of 33.78%. Among the ORFs, 55 commence with the initiation codon ATG, representing 82.09% of all ORFs, 8 start with TTG (11.94%), and 4 start with GTG (5.97%). Annotation of the ORFs using the NCBI NR database revealed that 61 ORFs matched homologous gene families, constituting 91.04% of the total. Out of these, 31 ORFs are predicted to have potential gene functions. They are classified into functional categories, such as DNA replication, regulation, lysogeny, host lysis, phage DNA packaging, and phage morphogenesis. Notably, annotations for 29 ORFs are associated with *Staphylococcus* phages, while the remaining 38 are linked to *Caudoviricetes* phages.

**Figure 3:**
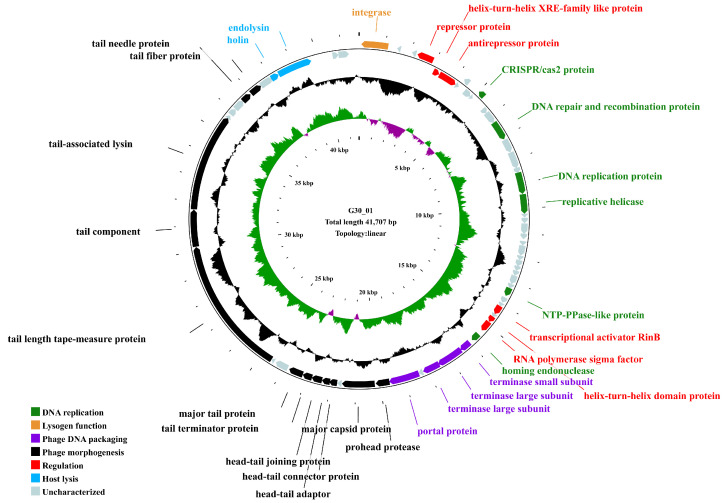
Genomic map of vB_G30_01. The outermost circle represents the open reading frames (ORFs), with distinct colors indicating various functional categories. The second circle, depicted in black, represents the GC content. The third circle displays GC skew (green for values greater than 0, purple for values less than 0), and the innermost circle denotes the scale of the genome size.

The genome of vB_G30_01 encompasses genes for various proteins, including an integrase protein (ORF1), a repressor protein (ORF4), and an anti-repressor protein (ORF6). Additionally, it features a terminase small subunit (ORF40) and two terminase large subunits (ORF41 and ORF42). The phage’s structural proteins consist of major capsid protein (ORF46), head–tail connector protein (ORF48), head–tail adapter (ORF49), head–tail joining protein (ORF50), tail terminator protein (ORF51), major tail protein (ORF52), tail length tape measure protein (ORF55), tail component (ORF56), and tail-associated lysin (ORF57). The genomic map (Figure 3) displays other encoded functional proteins, including 36 hypothetical proteins with unannotated functions. No tRNA genes, resistance genes, or virulence factors were identified in the vB_G30_01 genome using tRNAscan-SE, CARD, and VFDB databases.

### 3.3. Comparative Genomic Analysis

**Figure 4:**
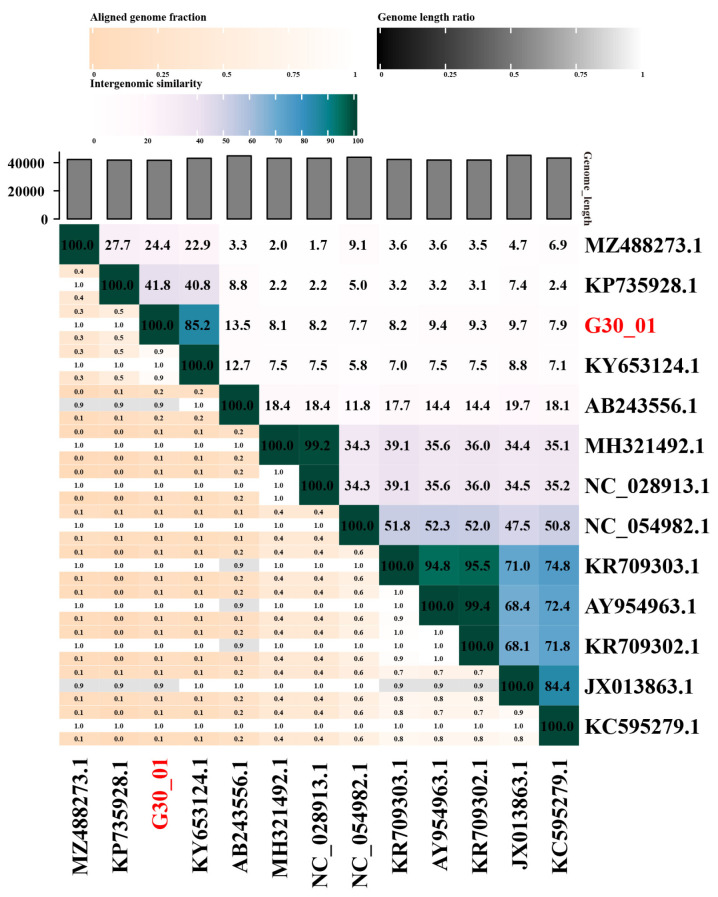
Comparison results of phage sequence similarity calculated using VIRIDIC. The axis labels indicate the Genbank accession numbers of the phages, with the phage name from this study highlighted in red font.

The VIRIDIC software v1.1 was utilized to calculate the genomic similarity between vB_G30_01 and related phages in the database, as depicted in the heatmap (Figure 4). The results revealed that vB_G30_01 exhibited the highest genomic similarity, of 85.2%, with *Staphylococcus* phage IME1367_01 (KY653124.1), followed by phage IME-SA4 (KP735928.1) at 41.8%, phage vB_SarS_BM31 (MZ488273.1) at 24.4%, and phage phiPVL108 (AB243556.1) at 13.5%. The remaining phages displayed less than 10% similarity.

Furthermore, a BLASTn search of the vB_G30_01 complete genome sequence against the NCBI nucleotide collection (NR/NT) database revealed that phage vB_G30_01 exhibited the highest similarity of 99.98% with *Staphylococcus* SWO (CP033098.1), achieving 100% query coverage. vB_G30_01 displayed a maximum similarity of 99.06% with *Staphylococcus* phage IME1367_01 (KY653124.1), with a query coverage of 84%. *Staphylococcus haemolyticus* phage IME-SA4 (KP735928.1) achieved a query coverage of 40%, with a similarity of 77.42%, and *Staphylococcus arlettae* phage vB_SarS_BM31 (MZ488273.1) had a coverage of 18%. For the remaining alignment results, coverage rates for samples were below 10% or belonged to sequences of uncultured viruses.

**Figure 5:**
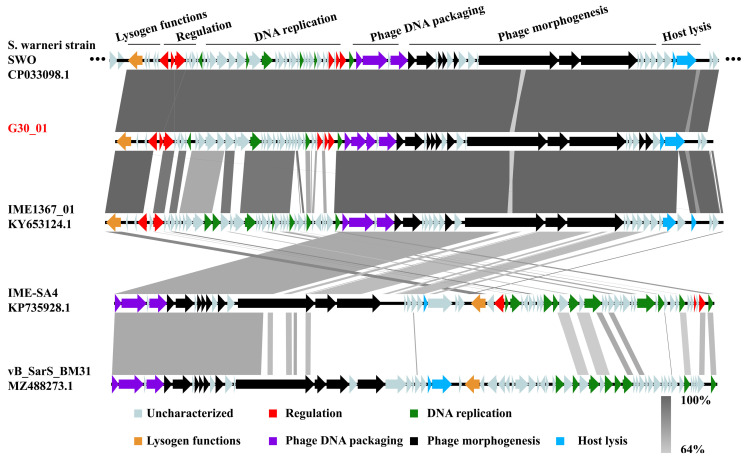
Comparative genomic analysis of the prophage region in *S. warneri* SWO, *S. warneri* phages vB_G30_01 and IME1367_01, *S. haemolyticus* phage IME-SA4, and *S. arlettae* phage vB_SarS_BM31. Arrows depict coding sequences and their respective transcription directions, with colors indicating various predicted functions: lysogenic functions (orange), transcription regulation (red), phage DNA packaging (purple), DNA replication (green), phage morphogenesis (black), and host lysis (blue). Gray arrows represent ORFs with unknown functions. The percentage of nucleotide sequence similarity is illustrated by varying shades of gray bars.

The prophage regions within the bacterial genome of *Staphylococcus warneri* strain SWO, as predicted by Phaster, were analyzed alongside four closely related phages (IME1367_01, IME-SA4, and vB_SarS_BM31) using Easyfig software v2.2.3 for comparative genomics, as depicted in Figure 5. The prophage region in the *Staphylococcus warneri* SWO genome exhibited high similarity to the genome of phage vB_G30_01, with the exception of genes associated with phage morphogenesis and lysis function. Phage IME1367_01 displayed substantial similarity to vB_G30_01 in lysogenic functions, phage DNA packaging, and phage morphogenesis but exhibited notable differences in genes related to transcription regulation and DNA replication. In contrast, IME-SA4 and vB_SarS_BM31 showed very low genomic similarities. Nevertheless, it is noteworthy that all phages exhibited over 60% similarity in regions related to phage DNA packaging and certain aspects of phage morphogenesis.

### 3.4. Phylogenetic Analysis

**Figure 6:**
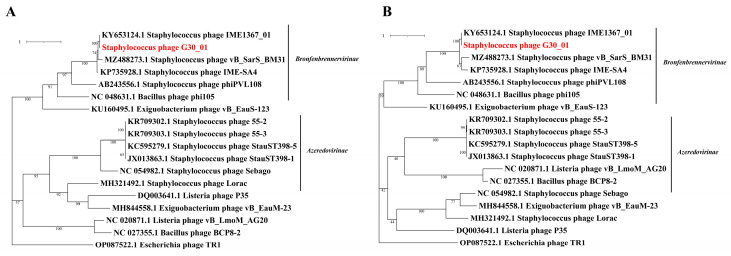
Phylogenetic trees of vB_G30_01 and related groups phylogenetic trees were constructed based on the amino acid sequences, focusing on the following two key components: (**A**) the terminase large subunit and (**B**) the major capsid protein. vB_G30_01 is distinctly highlighted in red for easy identification.

The phylogenetic tree was developed based on the amino acid sequences of crucial functional proteins from the following conserved areas: the major capsid protein and terminase large subunit in the phage. The phylogenetic tree included phages infecting representatives of the same bacterial family, with *E. coli* phage protein sequences as the outgroup (Figure 6). It is generally divided into two major branches, with vB_G30_01, *Staphylococcus* phages, *Bacillus* phages, and *Exiguobacterium* phages clustered in one branch, and the other branch grouping the aforementioned phages, along with *Listeria* phages.

## 4. Discussion

The genus *Staphylococcus* comprises nearly 50 species, with 38 fully identified as coagulase-negative staphylococci (CNS) [50]. The complexity of CNS species, in comparison to *Staphylococcus aureus*, results in variations in epidemiology, pathogenicity, virulence, ecology, host adaptability, and antibiotic resistance. Consequently, conducting genotypic and phenotypic studies on individual species becomes crucial [51]. In certain environments, the abundance of CNS significantly surpasses that of *Staphylococcus aureus* [52]. However, the available phage information for CNS in public databases is limited [53]. *Staphylococcus epidermidis* phages have been extensively reported, with over 10 types registered in databases [24,25,26,27,28,29], while phages of other CNS species are only sporadically documented [54,55,56,57,58,59,60]. It is worth noting that the NCBI database includes the genome of *Staphylococcus warneri* phage IME1367_01 (GenBank: KY653124.1), but it has not undergone extensive analysis. Consequently, there is an urgent need for research on CNS phages and the expansion of genomic databases to enhance our understanding in this domain.

In this study, phage vB_G30_01 was isolated from *Staphylococcus warneri* G30 induced with mitomycin C, originating from cherry rhizosphere soil. Under electron microscope observation, vB_G30_01 displays a long, flexible, noncontractile tail attached to a regular polyhedral head. Notably, the deeper color within the phage capsid suggests a lower density and substantial dye infiltration, which may be attributed to the incompleteness of phage maturation. This may also result in some phage particles having an incomplete assembly of heads and tails, remaining in a separate state, as well as distorted capsids that lack DNA and are a byproduct of negative staining. Typically, *Staphylococcus* phages feature an icosahedral capsid and a noncontractile tail that terminates with a baseplate structure. The capsid may present elongated or isometric forms, while the tail lengths vary from short (130 nm) to long (400 nm) [61]. This conforms to the characteristic features of long-tailed phages, which are specifically known as siphoviruses. In 2022, the International Committee on Taxonomy of Viruses (ICTV) underwent a significant revision of viral classification, abolishing the three morphology-based groups: *Podoviridae*, *Siphoviridae*, and *Myoviridae* [62]. Presently, all phages fall under *Caudoviricetes* classification, with further categorization requiring genomic comparison for defining specific groups.

To assess *Staphylococcus warneri* phage vB_G30_01’s capability to infect other host bacteria, a plaque assay was conducted to determine its host range. However, no plaques were observed in the assay, indicating the absence of a confirmed suitable host for the transmission of vB_G30_01. Further molecular biological methods involving the design of PCR and qPCR primers targeting the major capsid protein gene of vB_G30_01 were employed. The presence and increase in the copy number of the phage *mcp* were assessed to determine whether the phage adsorbs onto bacterial cells and replicates with them [32]. PCR results indicated that strains G28 and D103 did not harbor the similar *mcp* gene of vB_G30_01 in their genomes (Figure S2). After infection with phage vB_G30_01, the major capsid protein gene was found in both strains, suggesting that the phage had either adsorbed to or injected itself into the bacteria (Figure S1). As the lysogenic host of vB_G30_01, strain G30 enabled the phage to replicate with the host, thereby using the *mcp* gene expression trend as a positive control for phage replication. The results showed that *mcp* gene expression in strain G30 significantly increased within 9 h. Similarly, *mcp* expression in strains G28 and D103 increased significantly after 9 h by more than three times, showing the same trend as strain G30. Thus, this indicated that phage vB_G30_01 entered the cells and replicated. Both *Staphylococcus* and *Bacillus* belong to Gram-positive bacteria, and their similarly structured cell walls might be recognized by the phage. It is reported that the atypical wall teichoic acid (WTA) of *Staphylococcus aureus* is similar to that of other *Staphylococcus* species and even of other genera. A “glycocode” of WTA structures and WTA-binding helper phages permits horizontal gene transfer (HGT), even across long phylogenetic distances, thereby shaping the evolution of Gram-positive pathogens [63]. However, whether vB_G30_01 integrates into the host genome or plasmid or enters the cell as free DNA requires further experimental verification.

The majority of *Staphylococcus* phages retrieved from databases are temperate phages, with their double-stranded DNA genomes typically integrating into the host’s genome. These phages undergo lysis and release into the extracellular environment due to induction under certain conditions [61]. Based on genome size, they can be classified into three categories (Type I: <20 kb; Type II: 20–40 kb; Type III: >125 kb). These correspond to the three morphological classifications: podovirus, siphovirus, and myovirus [64]. In this study, the genome of phage vB_G30_01 is a dsDNA of approximately 41,707 bp in length, with ORFs comprising over 90%, indicating minimal coding gaps and high gene density (1.61 gene·kb^−1^). All of these characteristics are consistent with the genomic attributes of *siphoviruses* [64]. The functional classification of ORFs displays the modular structure characteristic of phages, with genes encoding similar functions generally adjacent to each other (Figure 3). These functions include DNA replication, regulation, lysogenic functions, host lysis, phage DNA packaging, and phage morphogenesis packaging, with instances of very short open reading frame fragments inserted among homologous genes within the same functional area. This indicates the chimeric nature of the phage genome, revealing that phage populations can undergo continuous genetic exchange through various recombination modes [65]. Such recombination modes greatly increase the diversity of phages. Two modules related to host lysis are located downstream in the genome, with almost all genes positioned on the forward strand of the phage, and only the integrase related to lysogenic function and the repressor protein inhibiting phage lysis are located on the reverse strand of all 67 predicted ORFs; less than half can be predicted to have homologous functional genes, indicating that many functional genes in *Staphylococcus* phages urgently require further characterization and also providing a significant source of genetic diversity.

In the genome of vB_G30_01, six ORFs are predicted to be involved in DNA replication-related functions (refer to Figure 3, Table S4). The phage also encodes genes for DNA repair and modification but lacks genes for DNA polymerase and primase. ORF11 encodes the CAS2 protein in the CRISPR system, a ribonuclease family associated with the CRISPR system in prokaryotic microorganisms. This protein is known to cleave ssRNA predominantly in uracil-rich areas and is commonly utilized for identifying phage spacer sequences [66]. ORF18, 19 encodes a DNA helicase, and ORF32 encodes a protein of the NTP-PPase-like family. Homologs of these proteins have been found in many phage genomes. The NTP-PPase possesses a MazG structural domain and belongs to the α-nucleoside triphosphate pyrophosphohydrolase family. This enzyme is thought to hydrolyze all non-canonical nucleoside triphosphates generated as metabolic byproducts into monophosphate derivatives. Thus, it plays a role in the function of monophosphate derivatives [67]. ORF39 encodes a homing endonuclease homolog protein. In phage genomes, HNH nuclease genes are typically located near terminase genes [68,69]. This juxtaposition is highly conserved and may facilitate homologous recombination and gene conversion processes [70]. In the genome of vB_G30_01, the homing endonuclease ORF39 is located near the terminase genes (ORF40, ORF41, ORF42), which is consistent with the description.

ORF41 and ORF42 are predicted to encode the terminase large subunit (terL) (Figure 3, Table S4). The terminase generally consists of two subunits: a small one for recognizing viral DNA (packaging sequence) and a large one with nuclease and ATPase activity [70,71]. vB_G30_01 possesses two parts of terminase large subunit, with overlapping regions that encode proteins. Generally, the detection rate of SNPs is greater than 99% when the sequencing depth reaches 50× or more, and this genome was sequenced to a depth of 64,000X, so it is almost certain that a base mutation has occurred. This redundancy might result from recoding. In phages, this frameshift controls the production of two proteins with overlapping sequences, using less genetic material to produce multiple distinct proteins [72,73]. The codon CUU is especially susceptible to frameshift occurrences [73]. According to the sequence alignment of ORF41 and ORF42 with the terL homologs, it is speculated that a +1 frameshift occurred at position 17535. The anticodon GAA, initially pairing with codon CUU, is believed to slide back one position in the ribosome complex to align with UUU (CUU UAC), resulting in a leucine covering four nucleotides, followed by the termination codon UAG. In dsDNA phages, frameshifts are relatively common in tail protein-related genes [74,75]. However, frameshifts in the terL are less reported [32]. Thus, the speculated frameshift in the terL of vB_G30_01 requires additional experimental validation.

Moreover, six ORFs encode various types of transcriptional regulators (Figure 3, Table S4). This exceeds the average number of two in ancestral phages [76], suggesting that vB_G30_01 may play a significant role in the transcriptional regulation of its host. ORF4 and ORF6 encode a repressor protein and an antirepressor protein, respectively. These genes, acting as a transcriptional repressor and its antagonist, can establish and maintain lysogeny in prophages. They prevent phage DNA replication or inhibit the expression of the repressor, thus regulating phage DNA replication and transcription, including shifting the prophage to a lytic cycle [77,78]. ORF5 and ORF37 encode proteins with a helix-turn-helix (HTH) domain, which belongs to an ancient transcriptional regulator family. They are involved in various biological processes, including cell proliferation and DNA transfer [79]. Additionally, they play a role in regulating bacterial oxidative tolerance and virulence, as well as resistance to extreme environments [80,81]. These domains identify homologous DNA binding sites according to their structural features [82]. HTH is also the structure for recognizing DNA binding sites in phage repressor proteins [83]. ORF35 is predicted to belong to the transcriptional activator RinB protein family, which comprises various *Staphylococcus aureus* phage RinB proteins and related sequences from their hosts. The int gene of *Staphylococcus* phage phi 11 is the sole gene responsible for the integration and recombination; both rinA and rinB are essential for activating the expression of the int gene [84]. ORF38 encodes an RNA polymerase sigma factor, which is a structural basis of promoter recognition by *Staphylococcus aureus* RNA polymerase [85]. This indicates that vB_G30_01 might have a role in controlling the DNA translation process in the host.

ORF1 encodes an integrase (Figure 3, Table S4), which is crucial for integrating the phage genome into the host genome [86]. Integrase is a member of the two primary families of site-specific recombinases. Based on their catalysis features, it can be classified as either a serine or tyrosine recombinase [87]. High similarities of ORF1 with the tyrosine-type integrase homologs in the NR and Conserved Domain databases showed that the integrase of vB_G30_01 belongs to the tyrosine recombinase. Tyrosine-type integrases cleave and rejoin single strands in pairs, forming a holliday junction intermediate, which is the most common type of integrase in prokaryotes [87].

Phages need to leave host cells and spread out in search of new targets. The main obstacle is the continuous network structure of peptidoglycan. Apart from filamentous phages, which can squeeze directly through the envelope, other phages must break down peptidoglycan to lyse cells [88]. All dsDNA phages produce a soluble cell wall lytic enzyme known as endolysin. However, endolysin lacks a secretion signal sequence. Thus, holins, which make the membrane permeable, are required to allow membrane proteins to lyse the peptidoglycan structure [89,90]. In the genome of vB_G30_01, ORF64 and ORF65 are predicted to encode holin and endolysin, respectively (see Figure 3, Table S4), suggesting that vB_G30_01 employs a host lysis strategy similar to most phages.

VIRIDIC was employed to assess the similarity between vB_G30_01 and other known phage genomes. The findings reveal that vB_G30_01 shares the highest similarity of 85.2% with *Staphylococcus warneri* phage IME1367_01. According to ICTV classification, both can be categorized in the same genus, specifically within *Bronfenbrennervirinae*. Furthermore, vB_G30_01 exhibits 41.8% genomic similarity to *Staphylococcus haemolyticus* phage IME-SA4, 24.4% to *Staphylococcus albicans* phage vB_SarS_BM31, and 13.5% to *Staphylococcus aureus* phage phiPVL108. This suggests that phages infecting hosts within the same genus display higher similarity. A comparative genomic analysis of vB_G30_01 also substantiates this observation. Additionally, it reveals that proteins associated with phage DNA packaging and adjacent morphologies remain highly conserved, even among various hosts. Reviews on phages underscore the fact that despite the high nucleotide diversity of the phage genome, certain protein structures exhibit relative conservation across phage families, particularly those related to phage morphologies and DNA packaging [91].

A phylogenetic tree was constructed for vB_G30_01 and related phages based on the major capsid protein and the terminase large subunit. The host bacterial categories that phage vB_G30_01 can infect were screened out, as well as the common bacterial categories. Phages with the highest similarity to vB_G30_01 were selected as representatives to build the phylogenetic tree. The phylogenetic tree can be broadly divided into two clusters, *Bronfenbrennervirinae* and *Azeredovirinae*. vB_G30_01 exhibits the closest homology with *Staphylococcus warneri* phage IME1367_01, sharing the same host. Subsequent to this are phages of the genus *Staphylococcus* belonging to *Bronfenbrennervirinae*, indicating that the higher the host homology, the higher the protein homology of the phage. Interestingly, the major caspid protein of phages from certain *Bacillus* and *Exiguobacterium* species show higher protein homology with vB_G30_01 than those from *Staphylococcus*. This could be indicative of phages’ capability to infect and horizontally transfer genes across species.

Revelations from the genome analysis of prophage vB_G30_01 have significantly expanded our understanding of *Staphylococcus warneri* prophages. Given the limited scope of the CNS phage database, numerous unidentified proteins await detailed characterization. Furthermore, additional research is needed to explore the potential implications of vB_G30_01’s capacity for cross-genera infection on the evolutionary dynamics and interaction mechanisms of host bacteria.

## 5. Conclusions

In this study, we isolated and characterized a novel *Staphylococcus warneri* phage, vB_G30_01, showcasing its potential for cross-species infection. The genomic analysis of vB_G30_01 unveiled lysogeny-related functional genes, categorizing it as a temperate phage. With less than half of the ORFs matching predicted functions, numerous unknown functional proteins were identified, acting as a source of genetic diversity for host bacteria. The genome is predicted to encompass four genes related to DNA replication and six related to transcription regulation, indicating the phage’s potential role in host transcriptional control and metabolic assistance. Comparative genomic analysis suggests that vB_G30_01 can be classified within *Bronfenbrennervirinae*. vB_G30_01 displays higher genomic homology, with phages infecting hosts of the same genus. Furthermore, even phages from different bacterial genera exhibit conservation in proteins related to phage DNA packaging and morphogenesis. A phylogenetic analysis based on capsid proteins and terminase large subunits reveals close homology with phages infecting *Bacillus*, *Micrococcus*, and *Staphylococcus*. The genome’s comparative analysis, which is induced by bacteria, serves as an effective approach to studying whether phages participate in host metabolic regulation and adaptive changes.

## Data Availability

The genome sequence of vB_G30_01 used for this study is available in Genbank under GenBank accession number PP213047.

## Supplementary Materials

The following supporting information can be downloaded at https://www.mdpi.com/article/10.3390/v16101631/s1.
