# Supplementary figures and images for: Deciphering the Genetic Architecture of Staphylococcus warneri Prophage vB_G30_01: A Comprehensive Molecular Analysis
Source: Viruses. 2024 Oct 19;16(10):1631. doi: 10.3390/v16101631 (PMC11512304; doi:10.3390/v16101631)

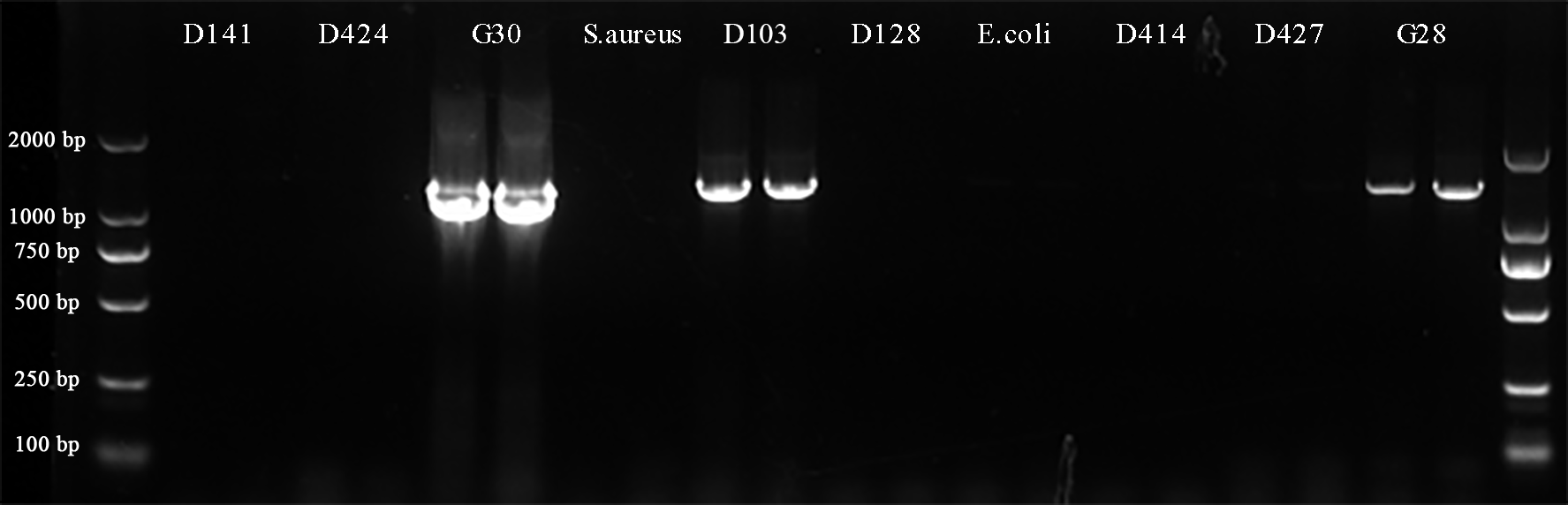

Supplement: Supplementary file 1 [file viruses-16-01631-s001.zip › Figure S1.tif]

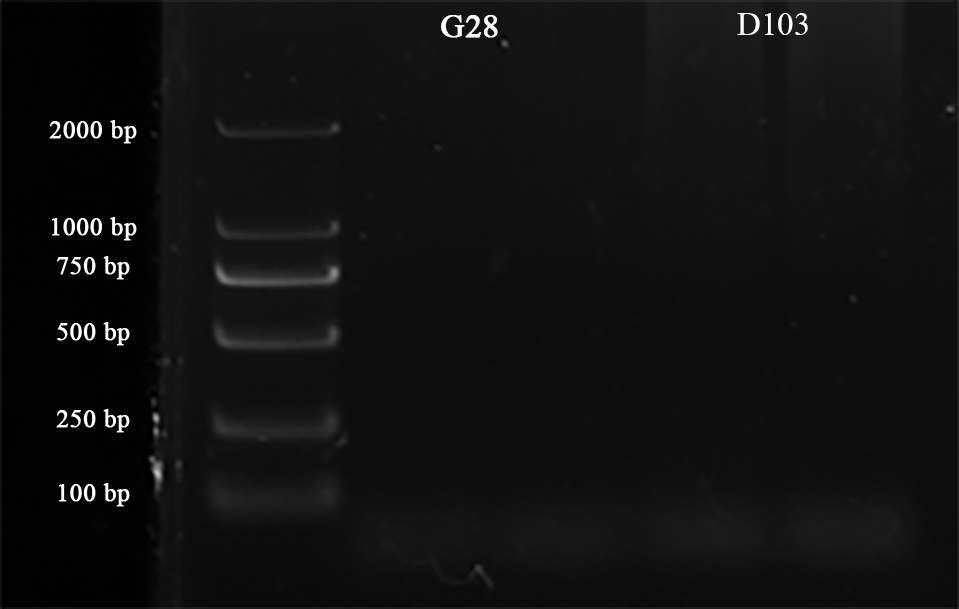

Supplement: Supplementary file 1 [file viruses-16-01631-s001.zip › Figure S2.tif]

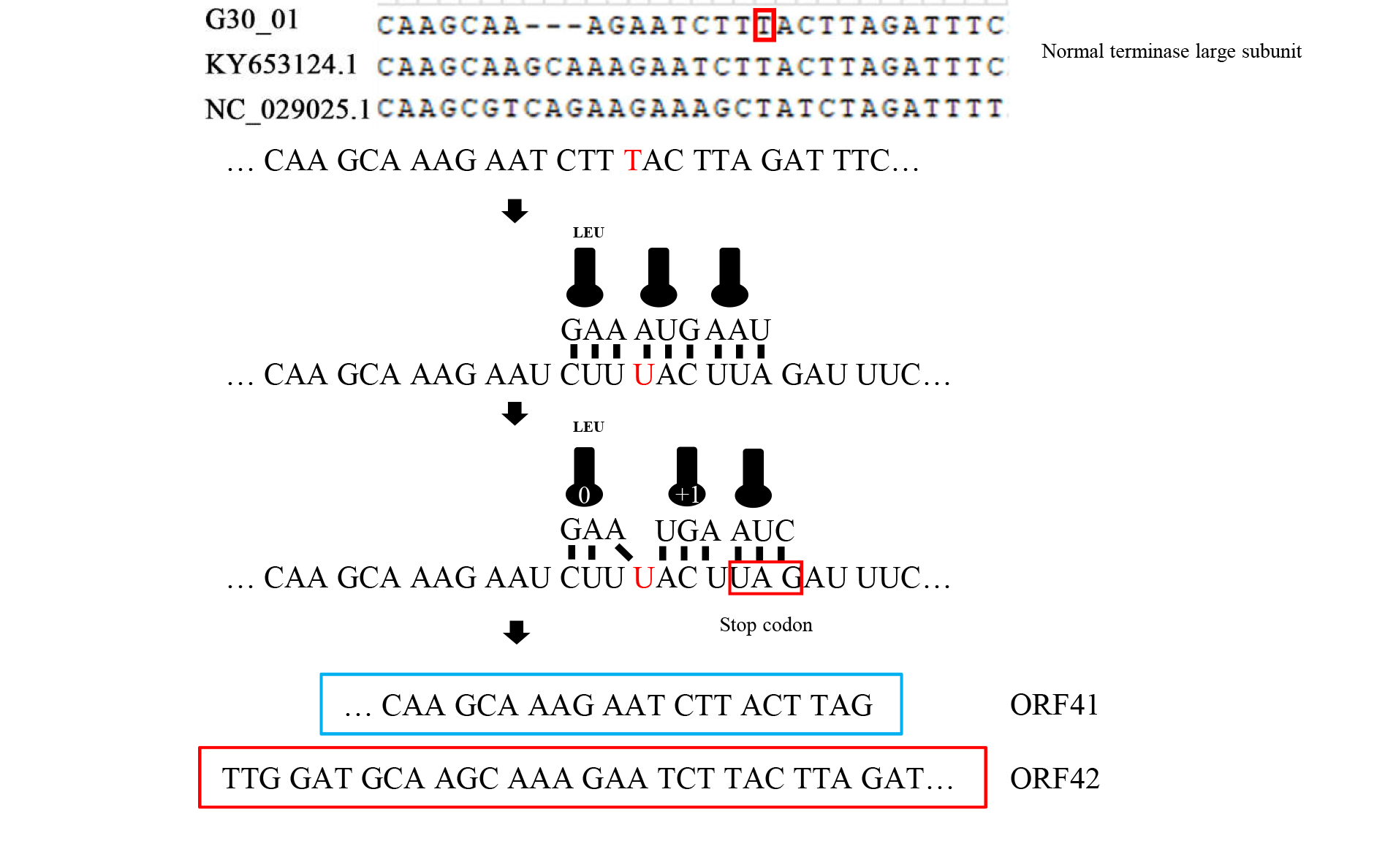

Supplement: Supplementary file 1 [file viruses-16-01631-s001.zip › Figure S3.tif]
